# Association between *Toxoplasma gondii* Infection and Type-1 Diabetes Mellitus: A Systematic Review and Meta-Analysis

**DOI:** 10.3390/ijerph20054436

**Published:** 2023-03-02

**Authors:** Ashley Catchpole, Brinley N. Zabriskie, Pierce Bassett, Bradley Embley, David White, Shawn D. Gale, Dawson Hedges

**Affiliations:** 1 The Neuroscience Center, Brigham Young University, Provo, UT 84602, USA; 2 The Department of Statistics, Brigham Young University, Provo, UT 84602, USA; 3 The Department of Micro and Molecular Biology, Brigham Young University, Provo, UT 84602, USA; 4 The Department of Psychology, Brigham Young University, Provo, UT 84602, USA

**Keywords:** type-1 diabetes mellitus, type-1 diabetes juvenile-onset diabetes, toxoplasmosis, *Toxoplasma gondii*

## Abstract

Type-1 diabetes, an autoimmune disease characterized by damage to pancreatic insulin-producing beta cells, is associated with adverse renal, retinal, cardiovascular, and cognitive outcomes, possibly including dementia. Moreover, the protozoal parasite *Toxoplasma gondii* has been associated with type-1 diabetes. To better characterize the association between type-1 diabetes and *Toxoplasma gondii* infection, we conducted a systematic review and meta-analysis of published studies that evaluated the relationship between type-1 diabetes and *Toxoplasma gondii* infection. A random-effects model based on nine primary studies (total number of participants = 2655) that met our inclusion criteria demonstrated a pooled odds ratio of 2.45 (95% confidence interval, 0.91–6.61). Removing one outlying study increased the pooled odds ratio to 3.38 (95% confidence interval, 2.09–5.48). These findings suggest that *Toxoplasma gondii* infection might be positively associated with type-1 diabetes, although more research is needed to better characterize this association. Additional research is required to determine whether changes in immune function due to type-1 diabetes increase the risk of infection with *Toxoplasma gondii,* infection with *Toxoplasma gondii* increases the risk of type-1 diabetes, or both processes occur.

## 1. Introduction

Type-1 diabetes is an autoimmune disease characterized by autoimmune damage to pancreatic insulin-producing beta cells. The physiology involves CD4+ T cells, CD8+ T cells, and macrophages, resulting in decreased insulin release and elevated glucose concentration in the blood [1]. Type-1 diabetes affects approximately nine million people worldwide [2] with substantial regional variation in prevalence [1]. The estimated prevalence of type-1 diabetes in the United States at age 18 years is 1 in 300 [3], and type-1 diabetes accounts for approximately five to ten percent of all cases of diabetes [3]. Type-1 diabetes is associated with a variety of adverse outcomes, including retinal, kidney [4], and cardiovascular diseases [3]. Further, type-1 diabetes is also associated with cognitive impairments and possibly dementia [5], making this autoimmune disease an important adverse factor in cognitive health. For unclear reasons, the incidence of type-1 diabetes is increasing in many regions [3]. Both genetic and environmental factors appear to be involved in the etiology of type-1 diabetes, including variants in human leukocyte antigens and multiple genetic loci [1].

Exposure to several types of infectious diseases is associated with type-1 diabetes [6], including viral infections, such as rubella, rotavirus, and enteroviruses [1]. An early study that evaluated exposure to five different infectious diseases and type-1 diabetes found that, while exposure to one infection was not associated with subsequent risk of diabetes, exposure to two (odds ratio: 2.274) and more than two (odds ratio: 5.798) infections was associated with a subsequent risk of diabetes [7]. Also associated with type-1 diabetes in some studies is the apicomplexan neurotropic protozoan *Toxoplasma gondii* [8,9], a parasite that affects approximately one-third of the world’s population [10] but with marked variance in seroprevalence and virulence [11] across the world. While some evidence indicates possible associations between *Toxoplasma gondii* infection and other autoimmune diseases [12], the association between *Toxoplasma gondii* infection and type-1 diabetes remains unclear. A previous meta-analysis of three studies that was published in 2016 [13] found no association between *Toxoplasma gondii* infection and type-1 diabetes (95% confidence interval, 0.13–9.57). However, a more recent study conducted in 2021 strongly supports a positive association between type-1 diabetes and *Toxoplasma gondii* [8]. In a murine model, Nassief Beshay et al. [14] found that, although *Toxoplasma gondii* infection did not result in diabetes, there was an increased prevalence of apoptotic indicators in the islets of Langerhans and a corresponding reduction in insulin expression with an increase in serum glucose concentration.

*Toxoplasma gondii* infection could affect the prevalence of type-1 diabetes via several mechanisms. The possible association between type-1 diabetes and *Toxoplasma gondii* infection could be due to the decreased immune function found in type-1 diabetes [15], thus leading to increased susceptibility to infection with *Toxoplasma gondii*. On the other hand, *Toxoplasma gondii* infection could induce autoimmunity with autoantibodies or other immunologic mechanisms, thus decreasing insulin secretion from pancreatic beta cells [16].

Given the large number of people with type-1 diabetes, the disease’s associated morbidity, and the large number of people worldwide who are seropositive for infection with *Toxoplasma gondii*, we sought to quantitatively characterize the association between *Toxoplasma gondii* and type-1 diabetes with a systematic review and meta-analysis based on currently available primary studies. Since the 2016 meta-analysis of three studies on the association between *Toxoplasma gondii* infection and type-1 diabetes [13], several new studies that evaluated this association were published, which we include here in an updated meta-analysis. In addition to increased available evidence, we also use different statistical methods that are more appropriate for a meta-analysis based on a relatively small number of studies. These two reasons, supported by recommendations from the Cochrane Collaboration on when to update a meta-analysis [17], prompted us to conduct an updated meta-analysis and review. The benefits of updating this meta-analysis are many. For example, by updating this analysis with additional studies, the results may enhance generalizability due to a larger number of independent samples in the various studies. Any additional studies would also increase the total number of participants both with and without type-1 diabetes, increasing the statistical power to determine whether there is an association between type-1 diabetes and *Toxoplasma gondii* infection. Further, applying the recommended statistical methods provides more reliable results on which to base the conclusions.

## 2. Methods

### 2.1. Information Source and Search Strategy

Using the PubMed electronic database, we searched for published articles that contained findings regarding the associations between type-1 diabetes and *Toxoplasma gondii*. We used the search terms “type-1 diabetes mellitus”, “juvenile-onset diabetes mellitus”, “diabetes mellitus”, “toxoplasmosis”, “*Toxoplasma gondii*”, and “*Toxoplasma*”. We also manually searched the reference lists of the included articles to identify other potential sources.

### 2.2. Article Selection Criteria

**Figure 1:**
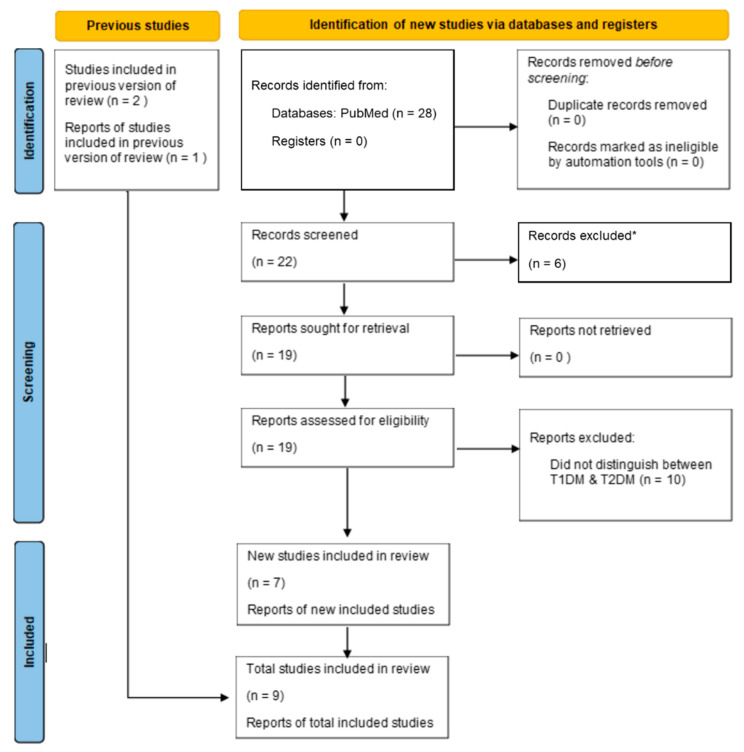
Flow chart of study selection. * Records excluded did not contain a control group of healthy individuals to compare with those infected with *Toxoplasma gondii* and type-1 diabetes mellitus. All six records were excluded by a human. T2DM = type-2 diabetes mellitus.

Searching up to and through November 2022, we included peer-reviewed published studies in any language that contained data on the seroprevalence of *Toxoplasma gondii* infection in a group with type-1 diabetes and an otherwise healthy control group. We excluded case studies and studies that did not include an otherwise healthy control group (Figure 1). In their 2016 meta-analysis, Majidiani et al. [13] included data from Abdelsalam, 2013. However, we and our institution’s research librarians were unable to locate Abdelsalam, 2013. Additionally, the corresponding author of Majidiani et al. [13] failed to respond when we asked for their Abdelsalam, 2013 data. As such, we were unable to include these findings in our systematic review and meta-analysis.

### 2.3. Data Extraction

Three trained members of our research group independently extracted the last name of the first author, the publication date, the region or country where the study was completed, the number of participants in the type-1 diabetes group and the control group, the number of participants seropositive for *Toxoplasma gondii* infection, the percent female of the participants in the type-1 diabetes group and the control group, the mean age of the participants in the type-1 diabetes group and control group, and the type of assay used to determine *Toxoplasma gondii* seropositivity. They then met together to identify, resolve, and correct any discrepancies in the extracted data. For articles published in languages other than English, we reviewed the articles potentially meeting our inclusion criteria with either a native speaker or a person proficient in the language to extract the relevant data. After extracting the data, we also reached out to the corresponding authors of each paper and requested any information that was not included, such as the percent female and mean age. Only one corresponding author responded with the percent female and mean age of the control group of their study.

### 2.4. Data Analysis

We conducted a meta-analysis using the meta package from the R software (version 4.2.2) to summarize the relationship between *Toxoplasma gondii* infection and type-1 diabetes [18,19]. We created forest plots to visualize the individual study odds ratios (OR), corresponding 95% confidence intervals (CIs), and heterogeneity among the studies. We formally assessed heterogeneity using the Cochran’s Q test and I^2^ statistics.

For the meta-analysis, we used the random-effects Mantel–Haenszel method. Various heterogeneity estimators were applied, including the DerSimonian–Laird, Paule–Mandel, restricted maximum likelihood, and Sidik–Jonkman estimators. The estimates of the heterogeneity variance ranged from 0.62 to 0.87; we report the most conservative results (obtained from the Sidik–Jonkman estimator). The confidence interval of the pooled odds ratio was constructed as outlined by Hartung and Knapp [20] and Sidik and Jonkman [21] to better reflect the uncertainty in the between-study heterogeneity estimation. Due to the small number of studies included in the analysis, we did not formally test for publication bias. We also performed sensitivity analyses to determine the impact of certain individual studies. We did not run a meta-regression due to the relatively small number of source studies.

## 3. Results

We screened 22 abstracts that potentially met our inclusion criteria, from which we reviewed 19 full papers. From these, nine studies met our inclusion criteria (Figure 1). From the nine studies meeting our inclusion criteria, 1219 participants were in the type-1 diabetes group, and 1436 participants were in the healthy control group for an overall total of 2655 participants. The percentage of *Toxoplasma gondii* seropositivity ranged from 8.8% to 86.4% in the type-1 diabetes group compared to 4.5% to 60% in the control group. Six of the studies were conducted in the Middle East, two in Asia, and one in South America. Not all source studies reported the average age and sex of the participants (Table 1).

The percentage of female participants in the type-1 diabetes group was reported across all studies but was omitted in the control group of two studies. The type of assay used to determine *Toxoplasma gondii* seropositivity varied across the studies, though four studies used the same enzyme-linked immunosorbent assay (ELISA). Seven of the nine source studies reported a positive association between *Toxoplasma gondii* infection and type-1 diabetes with *p* values ranging from <0.0001 to 0.042. Krause et al. [22] found a negative (protective) association between *Toxoplasma gondii* and type-1 diabetes, while Khalili et al. [25] found no significant association between the two.

**Figure 2:**
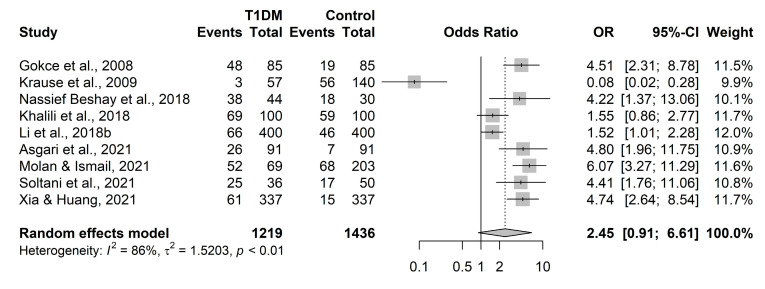
Meta-analysis results of the association between *Toxoplasma gondii* infection and type-1 diabetes (T1DM) using all nine identified studies [8,9,14,22,23,24,25,26,27]. OR, odds ratio; CI, confidence interval.

Figure 2 depicts a forest plot summarizing the nine individual study results, information regarding heterogeneity, and the pooled meta-analysis results. Strikingly, we saw markedly different results from the Krause et al. [22] study compared to the other eight studies. This, in part, contributed to substantial heterogeneity (Cochran’s Q test *p* < 0.0001 and I^2^ = 86%; I^2^ values above 75% often indicate substantial heterogeneity). The Mantel–Haenszel random-effects meta-analysis using the Sidik and Jonkman heterogeneity estimator produced a pooled odds ratio of 2.45 (95% CI of 0.91–6.61, *p* = 0.071, Figure 2).

While the lower bound of the 95% confidence interval is below one, which indicates no difference in the odds of *Toxoplasma gondii* seropositivity between the two groups, it is not much smaller than one, and the range of likely odds ratios is mostly positive. This suggests the odds of *Toxoplasma gondii* seropositivity may be higher for those with type-1 diabetes compared to healthy controls, though additional evidence is needed to substantiate this relationship.

**Figure 3:**
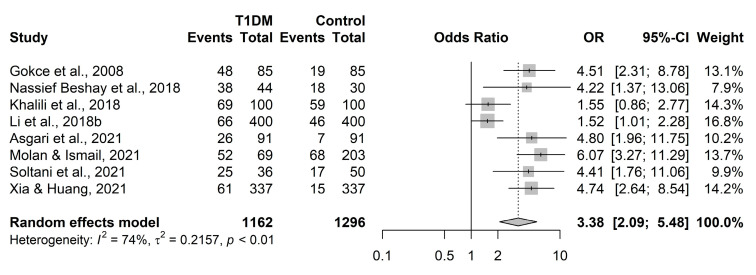
Meta-analysis results of the association between *Toxoplasma gondii* infection [8,9,14,23,24,25,26,27] and type-1 diabetes (T1DM) with Krause et al. [22] removed. OR, odds ratio; CI, confidence interval.

However, these overall results were mitigated by the Krause et al. [22] study, although the reason why the Krause et al. [22] study results differed from the results of the other source studies was never resolved due to insufficient data to run meta-regressions. Given that the Krause et al. [22] study resulted in a markedly different odds ratio and corresponding 95% confidence interval than the other studies, we performed a sensitivity analysis by conducting another meta-analysis with the Krause et al. [22] study removed simply to determine the effect this one study had on the meta-analysis results. While there was still considerable heterogeneity present (Cochran’s Q test *p* = 0.0004, I^2^ = 74%) as expected, the pooled odds ratio increased to 3.38 with a 95% confidence interval of 2.09–5.48 and *p* = 0.0006 (Figure 3), suggesting that the odds of *Toxoplasma gondii* seropositivity in this case are likely between 2.09 and 5.48 times higher for those with type-1 diabetes compared to healthy controls. Given the sparse reporting of important study-level characteristics and the small number of source studies, it is difficult to determine what aspects of the Krause et al. [22] study led to such different results from the other eight studies.

## 4. Discussion

The primary finding of this meta-analysis based on nine source studies is that the odds of *Toxoplasma gondii* seropositivity are likely between 0.91 and 6.61 times higher for those with type-1 diabetes compared to healthy controls (results obtained using a 95% level of confidence). Given that this interval overlaps the null value of one, the evidence that those with type-1 diabetes are more likely to be seropositive for *Toxoplasma gondii* is not overwhelming, though it is certainly suggestive. Removal of the one source study that provided markedly different evidence than did the other eight studies increased the odds ratio of *Toxoplasma gondii* seropositivity to between 2.09 and 5.48 higher for those with type-1 diabetes compared to healthy controls. It is unclear why Krause et al. [22] produced such contrasting conclusions compared to the other studies, as we were unable to fully understand the reasons behind this disparity. Although in the sensitivity analysis we removed the one article that found a negative association between *Toxoplasma gondii* infection and type-1 diabetes, we continued to include the one study that found no association either positive or negative. Overall, the findings from these meta-analyses suggest a possible positive association between *Toxoplasma gondii* seropositivity and type-1 diabetes, a hypothesis that requires additional study. These results differ from the previous meta-analysis that examined this relationship [13]. The previous meta-analysis contained only three primary studies that examined the association between *Toxoplasma gondii* infection and type-1 diabetes and resulted in a 95% confidence interval of the odds ratio of 0.13–9.57. As a result, Majidiani et al. [13] did not find an association between *Toxoplasma gondii* infection and type-1 diabetes, whereas we found a possible association between *Toxoplasma gondii* infection and type-1 diabetes.

Several reasons could account for the different findings between the previously published meta-analysis [22] and ours. Since the publication of the previous meta-analysis, several additional studies are now available. Whereas Majidiani et al. [13] were only able to include three primary studies, the availability of several more recent studies enabled us to include nine primary studies in our meta-analysis. Of the three studies in the Majidiani et al. [11] meta-analysis, two demonstrated positive associations between *Toxoplasma gondii* infection and type-1 diabetes, but one negative study rendered the overall conclusion of no significant relationship between the two. Additionally, we used different statistical methods to combine the source studies than did Majidiani et al. [13], which are recommended in practice for meta-analyses such as this [20,28,29].

While our study is not designed to identify the mechanisms by which *Toxoplasma gondii* infection could be associated with type-1 diabetes, at least a few broad and not necessarily mutually exclusive possibilities could account for a possible positive association between type-1 diabetes and *Toxoplasma gondii* infection. The immunocompromised state associated with type-1 diabetes [30] could increase *Toxoplasma gondii* infection rates. Neutrophil, monocyte, and T-cell dysfunction in type-1 diabetes could increase a host’s chances of contracting an infection with *Toxoplasma gondii*, as both the innate and adaptive immune responses may be impaired in type-1 diabetes [31]. With less neutrophil chemotaxis, the body is unable to fight off the initial infection with *Toxoplasma gondii* [32]. More than 25 percent of patients with type-1 diabetes lack complement component 4 (C4), a protein found on the surface of cells that is crucial in the opsonization of pathogens [33]. Without C4 marking the surface of a cell for phagocytosis, the parasite, in this case *Toxoplasma gondii*, is not neutralized, and infection is much more likely. In addition to these factors, there are several common abnormalities of the immune system before the onset of type-1 diabetes, including the depletion of memory CD4+ cells and defective natural killer-cell activity [33], which could increase the ability of *Toxoplasma gondii* to infect a host.

In addition to the decreased immune function associated with type-1 diabetes, another potential explanation for a possible positive association between *Toxoplasma gondii* infection and type-1 diabetes is that *Toxoplasma gondii* could induce autoimmunity against beta cells in the pancreas, thereby causing type-1 diabetes by damaging insulin secretion from beta cells. Prandota [34] argues that infection with *Toxoplasma gondii* can occur during pregnancy or shortly after birth, as maternal T cells and B cells transfer from mother to child. These T cells and B cells and their interactions with lymph nodes in the mesentery might then play a role in the development of type-1 diabetes. As demonstrated by Prandota [34], the offspring of a mother infected with *Toxoplasma gondii* during pregnancy, or shortly thereafter, had much higher rates of microchimerisms, genetically distinct cells from the mother, in their blood, consistent with the possibility that the mother’s infected cells then result in type-1 diabetes in the child. There is also the possibility that the spread of *Toxoplasma gondii* infected cells occurs in the fetus’ swallowing of amniotic fluid and during breastfeeding. The possibility of prenatal and perinatal infection with *Toxoplasma gondii* affecting pancreatic beta cells is consistent with theories that autoimmunity may be triggered in fetal life when the immature immune system is vulnerable and when tolerance and intolerance to antigens are more easily induced [35].

In a murine model, Oz [36] found that animals experimentally infected with *Toxoplasma gondii* developed necrotic lesions in their pancreases. They concluded that the death of pancreatic cells leads to the inhibition of insulin secretion and, therefore, the development of type-1 diabetes. Similarly, Nassief Beshay et al. [14] found that, in a murine model, toxoplasmosis increases susceptibility to developing type-1 diabetes due to the ability of the parasite *Toxoplasma gondii* to invade and replicate inside pancreatic cells, inhibit insulin production, and increase glucose concentration. Furthermore, Nassief Beshay et al. found that all mice with chronic *Toxoplasma gondii* infection had insulitis and inflammation of the islets of Langerhans [14]. There was also evidence of smaller islets and a change in the number of islets in chronically infected mice, possibly related to apoptosis.

Several factors require consideration in interpreting our findings. Only nine source studies met our inclusion criteria; however, these nine studies did contain a total of 1219 participants in the type-1 diabetes group and 1436 participants in the healthy control group. The comparatively small number of source studies renders our findings sensitive to findings from additional studies. Moreover, the small number of source studies precluded us from obtaining meaningful estimates of publication bias. Accordingly, we do not know how much our results could be due to negative studies not being published and, hence, unavailable for our meta-analysis (i.e., the file-drawer problem). In addition to the small number of studies, the source studies we used varied in how much demographic information they reported, resulting in too few demographic and other variables to perform meta-regressions aimed at identifying the sources of clinical and demographic heterogeneity between the source studies. The source studies were cross-sectional, thus precluding the drawing of causal inferences. We can only report a possible association between *Toxoplasma gondii* infection and type-1 diabetes. Given the morbidity associated with type-1 diabetes, the disease’s increasing incidence, and the high prevalence of *Toxoplasma gondii* infection, additional research is required to better understand any potential associations between type-1 diabetes and *Toxoplasma gondii* infection because, in part, *Toxoplasma gondii* infection could be a potentially modifiable risk factor. All source studies came from the Middle East, Asia, and South America, limiting the generalizability of our findings of a positive association between *Toxoplasma gondii* infection and type-1 diabetes in other regions and indicating the need to study the associations between *Toxoplasma gondii* infection and type-1 diabetes in different regions. Furthermore, there are different strains of *Toxoplasma gondii*, which can vary by region, and some of these strains are more virulent than others [11], making it important to investigate the associations between type-1 diabetes and *Toxoplasma gondii* infection in a variety of world regions with potentially different *Toxoplasma gondii* strains. As pathogenicity appears to differ between *Toxoplasma gondii* strains and lineages [11], it is possible that any association between type-1 diabetes and *Toxoplasma gondii* may depend, in part, on the strain of *Toxoplasma gondii*. Finally, given the cross-sectional nature of this data, it is impossible to know when a given participant was infected with *Toxoplasma gondii*. Although IgG antibodies suggest the infection is not acute, it is impossible to estimate when the infection occurred, if at any time it was reactivated, or if it did or did not occur during critical developmental periods in human growth. Prior work found, for example, that multiple childhood infections increase the risk for the development of type-1 diabetes [7]. Thus, it is currently unknown whether infection with *Toxoplasma gondii* confers a specific risk of development of type-1 diabetes or if the parasite simply acts as an additional infection that increases the risk along with other non-parasitic infectious pathogens. It is interesting that *Toxoplasma gondii* infection is also associated with type-2 diabetes, which has markedly different characteristics than type-1 diabetes in terms of purported cause, onset, and course [24].

## 5. Conclusions

The results of this meta-analysis of the nine primary studies that met our inclusion criteria demonstrate that the pooled odds ratio of *Toxoplasma gondii* seropositivity is 2.45 (95% CI of 0.91–6.61). The removal of an outlying study increased the pooled odds ratio to 3.38 (95% CI of 2.09–5.48). These findings suggest that *Toxoplasma gondii* infection might be positively associated with type-1 diabetes, although additional research is required to better characterize any possible association between type-1 diabetes and *Toxoplasma gondii* infection. This research must determine whether changes in the immune system due to type-1 diabetes could increase the risk of infection with *Toxoplasma gondii*, infection with *Toxoplasma gondii* could increase the risk of type-1 diabetes, or both processes could occur. Further, studies evaluating the associations between type-1 diabetes and *Toxoplasma gondii* infection from additional world regions are necessary because, in part, different areas could have different strains of *Toxoplasma gondii*, some of which could be more virulent than others. Investigations of whether demographic, medical variables, and sex influence the association between type-1 diabetes and *Toxoplasma gondii* infection are also necessary. Given the substantial morbidity associated with type-1 diabetes, the high prevalence of human *Toxoplasma gondii* seropositivity, and that infection with *Toxoplasma gondii* is potentially preventable, further research investigating the association between *Toxoplasma gondii* infection and type-1 diabetes is necessary.

## Figures and Tables

**Table 1:** Identified source studies comparing the percent of antibodies against *Toxoplasma gondii* infection in groups with and without type-1 diabetes.

Author, Ref.	T1DM Group				Control Group				*p* Value
	Cases, *n*	Female	Mean Age, Years	*T. gondii* Seropositive, *n*	Cases, *n*	Female	Mean Age, Years	*T. gondii.* Seropositive, *n*	
Krause et al. [22]	57	57.9%	16	3	140	-	-	56	0.001
Xia and Huang [23]	337	37.7%	40	61	337	-	-	15	<0.01
Soltani et al. [9]	36	47.2%	-	25	50	50%	-	17	0.001
Li et al. [24]	400	48.2%	-	66	400	44.8%	-	46	0.042
Nassief Beshay et al. [14]	44	59.1%	25	38	30	56.7%	29	18	0.027
Asgari et al. [8]	91	59.3%	-	26	91	63.7%	-	7	0.001
Khalili et al. [25]	100	50%	-	69	100	50%	-	59	NS
Gokce et al. [26]	85	47.1%	42	48	85	43.5%	42	19	<0.05
Molan and Ismail [27]	69	52.2%	37	52	203	49.3%	47	68	0.009

Dashes identify data not reported. T1DM = type-1 diabetes. *T. gondii* = *Toxoplasma gondii*. NS = not significant (the manuscript did not provide a *p*-value).

## Data Availability

No new data were created or analyzed in this study. Data sharing is not applicable to this article.
